# 5α-Reductase Type 2 Regulates Glucocorticoid Action and Metabolic Phenotype in Human Hepatocytes

**DOI:** 10.1210/en.2015-1149

**Published:** 2015-05-14

**Authors:** Maryam Nasiri, Nikolaos Nikolaou, Silvia Parajes, Nils P. Krone, George Valsamakis, George Mastorakos, Beverly Hughes, Angela Taylor, Iwona J. Bujalska, Laura L. Gathercole, Jeremy W. Tomlinson

**Affiliations:** Centre for Endocrinology, Diabetes and Metabolism (M.N., S.P., N.P.K., B.H., A.T., I.J.B.), Institute of Biomedical Research, School of Clinical and Experimental Medicine, University of Birmingham, Edgbaston, Birmingham B15 2TT, United Kingdom; Oxford Centre for Diabetes, Endocrinology & Metabolism (N.N., L.L.G., J.W.T.), NIHR Oxford Biomedical Research Centre, University of Oxford, Churchill Hospital, Headington, Oxford OX3 7LJ, United Kingdom; and Endocrine Unit, Second Department of Obstetrics and Gynecology and Pathology Department (G.V., G.M.), Aretaieion University Hospital, Athens Medical School, Athens, 11528, Greece

## Abstract

Glucocorticoids and androgens have both been implicated in the pathogenesis of nonalcoholic fatty liver disease (NAFLD); androgen deficiency in males, androgen excess in females, and glucocorticoid excess in both sexes are associated with NAFLD. Glucocorticoid and androgen action are regulated at a prereceptor level by the enzyme 5α-reductase type 2 (*SRD5A2*), which inactivates glucocorticoids to their dihydrometabolites and converts T to DHT. We have therefore explored the role of androgens and glucocorticoids and their metabolism by *SRD5A2* upon lipid homeostasis in human hepatocytes. In both primary human hepatocytes and human hepatoma cell lines, glucocorticoids decreased de novo lipogenesis in a dose-dependent manner. Whereas androgen treatment (T and DHT) increased lipogenesis in cell lines and in primary cultures of human hepatocytes from female donors, it was without effect in primary hepatocyte cultures from men. *SRD5A2* overexpression reduced the effects of cortisol to suppress lipogenesis and this effect was lost following transfection with an inactive mutant construct. Conversely, pharmacological inhibition using the 5α-reductase inhibitors finasteride and dutasteride augmented cortisol action. We have demonstrated that manipulation of *SRD5A2* activity can regulate lipogenesis in human hepatocytes in vitro. This may have significant clinical implications for those patients prescribed 5α-reductase inhibitors, in particular augmenting the actions of glucocorticoids to modulate hepatic lipid flux.

The global epidemic of obesity and type 2 diabetes is tightly linked to the increasing prevalence of nonalcoholic fatty liver disease (NAFLD), which contributes significantly to increased morbidity and mortality (1). The potent role of glucocorticoids (GC) to modulate metabolic phenotype is exemplified in patients with GC excess, Cushing's syndrome, and many of these patients develop NAFLD (2). However, in most patients with metabolic disease and NAFLD, circulating GC levels are not elevated (3). At a tissue-specific level, notably within the liver, GCs are cleared by a series of enzymes including the A-ring reductases (5α-reductase type 1 [*SRD5A1*] and 2 [*SRD5A2*] and 5β-reductase). 5α-reductase exists as 2 isoforms (*SRD5A1* and *SRD5A2*), both have five exons and four introns, but share less than 50% homology and both isoforms are expressed in human liver (4); *SRD5A1* alone is expressed in mouse liver. *SRD5A2* is believed to be the major isoform in clearing cortisol in human studies (5); however, there is an emerging role for *SRD5A1* in the pathogenesis of metabolic disease. We and others (6, 7) have shown that in a rodent model, genetic ablation of *SRD5A1* increase lipid accumulation in the liver and the severity of NAFLD.

The role of androgens in the pathogenesis of metabolic disease remains controversial. There is evidence documenting an association between hypogonadism and NAFLD (8, 9) with some evidence for improvement following androgen treatment (10, 11). *SRD5A2* has an established role in the conversion of T to DHT and genetic mutations lead to 46XY disorder of sex development. Although DHT is a more potent activator of the androgen receptor (*AR*), T binds and activates the *AR*. We have shown that increased global 5α-reductase activity is associated with impaired glucose tolerance and may be a future predictor of metabolic disease (12, 13). The lack of *SRD5A2* expression in the mouse liver (contrasting with human liver) has limited the interpretation of data from *SRD5A2* knockout mice (7) and has highlighted the importance of the use of human models. The translational importance of this not only relates to enhancing our understanding of the pathogenesis of NAFLD, but also to the widespread use of *SRD5A2* inhibitors including the selective, *SRD5A2* inhibitor, finasteride, and the nonselective (*SRD5A1* and *2*) inhibitor, dutasteride.

Lipid accumulation within hepatocytes is the first step in the development of NAFLD, and in some individuals can progress through inflammation to fibrosis and eventual cirrhosis. There are multiple mechanisms that contribute to lipid accumulation in vivo including re-esterification of free fatty acids delivered principally from intra-abdominal tissue depots, de novo synthesis of triacylglycerol from acetyl Coenzyme A (CoA) (de novo lipogenesis [DNL]) as well as limitation of β-oxidation and lipid export and secretion. Although free fatty acid delivery is believed to be the most important process in the development of NAFLD, the contribution of DNL increases significantly in patients with NAFLD (14). The rate-limiting step in DNL is the carboxylation of acetyl CoA to malonyl-CoA by acetyl CoA carboxylase (ACC), which is subsequently converted by a multistep reaction to palmitate by fatty acid synthase (FAS). There are two isoforms of ACC (ACC1 and ACC2); in lipogenic tissues ACC1 predominates and is the key regulatory step of fatty acid synthesis. ACC2 is localized to the mitochondrial membrane and its role is to limit β-oxidation through malonyl-CoA-mediated inhibition of carnitine palmitoyl transferase I.

Although it is not possible to replicate all the processes that contribute to the development of NAFLD using in vitro systems, using established cellular models, we have tested the hypothesis that *SRD5A2* represents an important regulator of the metabolic actions of androgens and GCs to modulate lipid homeostasis within human hepatocytes.

## Materials and Methods

### C3A and primary human hepatocyte culture

The C3A human hepatocyte cell line was purchased from LGC Standards (ATCC-CRL-10741), and cultured in Eagle's Minimum Essential Medium containing 10% fetal calf serum and glutamine/penicillin/streptococcus. Cells were seeded in 24-well plates and at 70–80% confluence were incubated with control media with or without hormonal treatments. The precise conditions for individual experiments is detailed in the results section. All reagents were supplied by Sigma-Aldrich unless otherwise stated.

Primary human hepatocytes were purchased from Celsis In Vitro Technologies. All donors were healthy, nondiabetic, none consumed alcohol above recommended limits (females, < 14 U/wk; males, < 21 U/wk), none were taking regular medications, and all had negative viral hepatitis serology (males, n = 4; age 54 ± 14 y; body mass index, 28.4 ± 3.3 kg/m^2^; females: n = 4; age 56 ± 4.7 y; body mass index, 23.98 ± 3.1 kg/m^2^). Cells were cultured overnight in Williams' Medium E without any supplements before being treated with GCs or androgens. For insulin-signaling studies, media was spiked with insulin 15 minutes prior to cell harvest as described above. Lipogenesis was measured by the uptake of 1-[14C]-acetate into the lipid component (see De novo lipogenesis).

### RNA extraction and reverse transcription

Total RNA was extracted from tissue and cells using the Tri-Reagent system. RNA integrity was assessed by electrophoresis on 1% agarose gel. Concentration was determined spectrophotometrically at OD_260_. In a 50-μL volume, 500 ng of total RNA was incubated with 250uM random hexamers, 500uM dNTPs, 20 U RNase inhibitor, 63 U Multiscribe reverse transcriptase, 5.5mM MgCl, and 1× reaction buffer. The reverse transcription reaction was carried out at 25°C for 10 minutes, 48°C for 30 minutes, and then the reaction was terminated by heating to 95°C for 5 minutes.

### Real-Time PCR

mRNA levels were determined using an ABI 7500 sequence detection system (Perkin-Elmer Applied Biosystems). Reactions were performed in 10-μL volumes on 96-well plates in reaction buffer containing 2× TaqMan Universal PCR Master mix (Applied Biosystems). All primers and probes were supplied by applied biosystems assay on demand (Applied Biosystems) and reactions normalized against the housekeeping gene 18S rRNA, provided as a preoptimized control probe. All target genes were labeled with FAM and the housekeeping gene with VIC. The reaction conditions were as follows: 95°C for 10 minutes, then 40 cycles of 95°C for 15 seconds and 60°C for 1 minute.

Data were obtained as ct values (ct, cycle number at which logarithmic PCR plots cross a calculated threshold line) and used to determine Δct values (Δct = ct of the target gene − ct of the housekeeping gene). Data were expressed as arbitrary units using the following transformation (expression = 1000 × [2^−Δct^] arbitrary units).

### Protein extraction and immunoblotting

Total protein was extracted from cells using RIPA buffer (50mM Tris pH, 7.4; 1% NP40; 0.25% sodium deoxycholate; 150mM NaCl; 1mM EDTA; 1mM PMSF and protease inhibitor cocktail [Roche] dissolved in 10 mL of distilled water) and freeze thawing. Protein concentrations were measured using a commercially available assay (Bio-Rad Laboratories Inc). Fifteen micrograms of protein was resolved on a 12.5% SDS-PAGE gel and transferred onto nitrocellulose membrane, Hybond ECL (GE Healthcare). Primary (PKB/akt, Biosource and anti phosphoPKB/akt [serine 473], R&D Systems) and secondary antibodies (Dako) used at a dilution of 1/1000. Membranes were reprobed for β-actin. Primary and secondary antibodies were used at a dilution of 1/5000 (Abcam). For antibody characteristics see Supplemental Table 1. Bands were quantified with Genesnap by Syngene and expressed relative to β-actin to normalize for gel loading.

### De novo lipogenesis

De novo lipogenesis (DNL) was measured by the uptake of 1-[^14^C] -acetate into the lipid component of hepatocytes as described previously (15). Cells were cultured in a 24-well plate, washed three times with serum-free media and then incubated with 500 μL of serum-free media with 4.44 kBq/L 1-[^14^C]-acetic acid with cold sodium acetate to a final concentration of 10μM acetate and treated with or without insulin (0.5 ng/mL). The cells were incubated at 37°C for 6 hours. After incubation activity was terminated by washing the cells three times with cold PBS and scraping into 250 μL of PBS. The lipid fraction was recovered in Folch solvent, the solvent was evaporated, and the radioactivity retained in the cellular lipid was determined by scintillation counting and expressed as disintegrations per minute/well. To account for variability between experimental replicates, data are presented as percentage change from control.

### β-oxidation

Rates of β-oxidation were measured by the conversion of [^3^H]-palmitate to ^3^H_2_O. Cells were cultured in a 24-well plates and were washed three times with serum-free media. Cells were then incubated with 300 μL of low glucose serum-free media with 4.44 kBq/L [^3^H]-palmitic acid with cold palmitate to a final concentration of 10μM. The cells were incubated at 37°C for 24 hours. After incubation medium was recovered and precipitated with an equal volume of 10% tricholoroacetic acid. The aqueous component of the supernatants was extracted with 2:1 choloform methanol solution. Radioactivity was determined by scintillation counting, expressed as disintegrations per minute/well and finally calculated as percentage change from control.

### Transfection studies

The Androgen receptor (*AR*) and *SRD5A2* cDNAs were cloned into the pcDNA3.1 vector (Invitrogen) and transiently transfected into C3A cells. Prior to transfection, cells were seeded into a 24-well plate. Cells were ∼60–70% confluent to obtain the most efficient transfection results. Transfection mixture comprised 1.5 μg of DNA diluted in 50 μL of OptiMEM serum-free media (Invitrogen) and 2 μL of Lipofectamine 2000 (Invitrogen) diluted in 50 μL of Optimem serum-free media. One hundred microliters transfection mixture was added dropwise to each well. The plates were rocked gently and left for incubation at 37°C. Transfection duration was 48 hours and its efficiency was determined by applying a plasmid containing the Green Fluorescent Protein (GFP) gene. Changes in *AR* and *SRD5A2* mRNA expression level were confirmed by real-time PCR.

### Site-directed mutagenesis

The R246Q mutant was inserted by site-directed mutagenesis into the *SDR5A2* cDNA using the Quikchange II site-directed mutagenesis kit (Agilent Technologies) as per the manufacture's guidance. In a 50-μL reaction the following components were added: 5 μl of 10× reaction buffer, forward and reverse primers (125 ng), 10 ng of double-strand DNA template, 1 μL of dNTP mix, 3 μL of QuikSolution, and ddH_2_O to a final volume of 50 μL. Using a thermal cycler (Biometra) samples were incubated at 95°C for 1 minute and then cycled 18 times at 95°C for 50 seconds, 53.4–60°C for 50 seconds, and 68°C for 60 seconds. Samples were then incubated for 68°C for 7 minutes. One microliter DpnI was added to PCR reaction, vortexed, and incubated for 1 hour at 37°C. One mililiter of ethanol (100%) was then added to each tube and incubated for 1 hour at −80°C. The mixture was centrifuged at 16 000 × *g* for 20 minutes at 4°C and the supernatant aspirated. The DNA pellet was washed with 75% ethanol, centrifuged, aspirated, air dried for 10 minutes, resuspended in 10 μL of RNase-free water. Finally, the DNA vector containing the R246Q mutation was transformed to XL10-Gold ultracompetent cells.

### Gas and liquid chromatography/mass spectrometry

Cortisol was extracted from cell media after addition of the internal standard cortisol-d4. Briefly, transfected cells were incubated with 200nM of cortisol for 24 hours. One milliliter of media was collected, extracted by SPE and the samples were derivatized overnight to form methyloxime trimethylsilylethers. The final derivative was dissolved in 55 μL cyclohexane, which was transferred to an autosampler vial for gas chromatography/mass spectrometry (GC/MS) analysis. An Agilent 5973 instrument (www.agilent.com) was used in a selected ion monitoring mode.

5α-reductase activity was measured using liquid chromatography/mass spectrometry (LC/MS). Briefly, cells were incubated with 100nM T for 30 minutes. Media was removed and transferred into glass tubes. Five milliliters dichloromethane was added to each tube, vortexed for 30 seconds, and then centrifuged for 10 minutes at 1600 rpm. The aqueous phase was removed and the steroid-containing organic solvent phase evaporated in air to dryness. The steroid extract was analyzed using LC-MS/MS (Xevo TQ mass spectrometer combined with an acquity uPLC system) with an electro-spray ionisation source in positive ion mode. Steroid hormones were eluted from a BEH C_18_ 2.1 × 50 mm 1.7 μm column using a methanol/water gradient system, solvent A was water 0.1% formic acid, and B was methanol 0.1% formic acid. The flow rate was 0.6 mL/min and starting conditions were 45% B increasing linearly to 75% B over 5 minutes. Steroid hormones were positively identified by comparison of retention times and mass transitions to steroid standards.

### Statistical analysis

Data are presented as mean ± SE. Where data were normally distributed, *t* tests (paired or unpaired where appropriate) were used to compare single treatments to control. If normality tests failed, nonparametric tests were used. ANOVA was used to compare multiple doses and/or treatments. Statistical analysis on real-time PCR data was performed on mean Δct values and not fold changes. All analysis was performed using the GraphPad Prism 6.0 software package (GraphPad Software, Inc).

## Results

### Regulation of lipogenesis human hepatocytes by androgens

**Figure 1.**
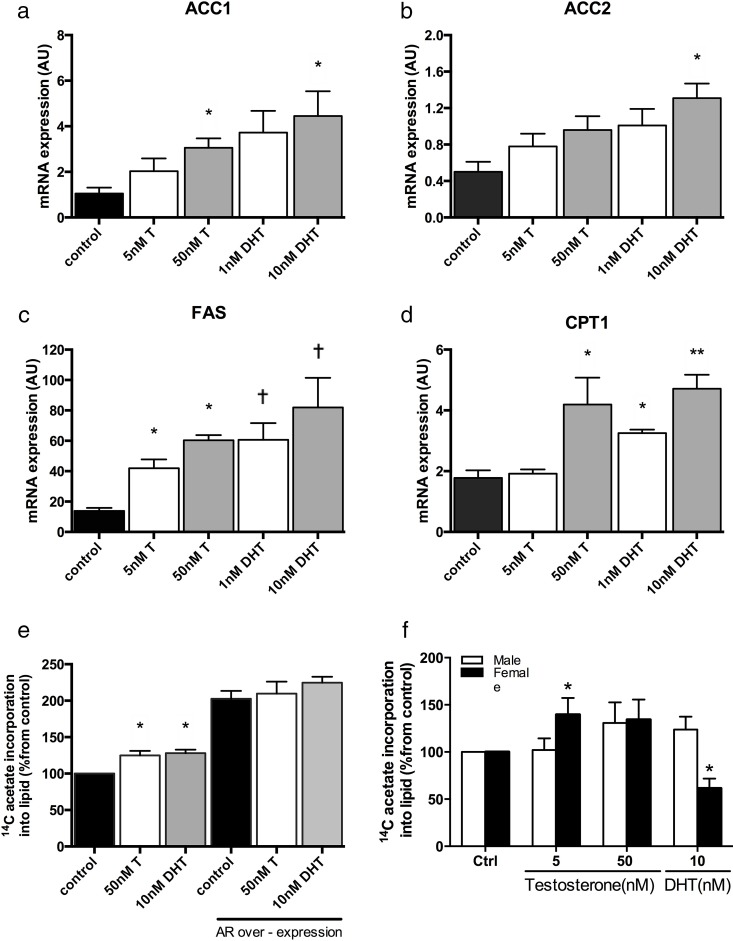
T and DHT cause a dose-dependent increase in lipogenic genes as well as increasing *carnitine palmitoyl trasferase 1 (CPT1)*, the rate-limiting step in mitochondrial β-oxidation, in human C3A cells (A–D). Changes in lipogenic gene expression are paralleled by function increases in 1-[^14^C]-acetate incorporation into lipid (E). *AR* overexpression alone and in the presence of T or DHT increases functional lipogenesis (E). In primary cultures of human hepatocytes, T but not DHT increases 1-[^14^C]-acetate incorporation into lipid in female patients (black bars), but have no effect in samples from male patients (white bars) (F). Data presented are mean ± SE; n = 3–5 experiments performed in triplicate; *, *P* < .05.

Fatty acid synthase (*FASN*), *ACC1*, *ACC2*, and carnitine palmitoyl transferase 1 (*CPT1*) mRNA expression increased after treatment with T in a dose-dependent manner (Figure 1, A–D) in C3A human hepatoma cells. Observations were similar following DHT treatment (Figure 1, A–D). Absolute changes in mRNA expression levels are presented in Supplemental Table 2. Lipogenic gene expression changes were mirrored by functional assays of 1-[^14^C]-acetate incorporation into lipid; both T and DHT increased lipogenesis. Lipogenic gene expression changes were mirrored by functional assays of 1-[^14^C]-acetate incorporation into lipid; both T and DHT increased lipogenesis (ctrl, 100% vs T, [50nM, 24 h] 124.9 ± 6.2%; DHT [10nM, 24 h] 128.1 ± 4.7%). *AR* overexpression was confirmed by real-time PCR (ctrl, 0.02 ± 0.003 vs *AR*, 30.04 ± 0.018 AU; *P* < .05). Even in the absence of T or DHT, *AR* overexpression alone caused a significant increases in 1-[^14^C]-acetate incorporation into lipid (ctrl vector only, 100% vs *AR*, 202.7 ± 10.8%; *P* < .05). Treatment with both T and DHT did not further enhance lipid accumulation (ctrl, 202.7 ± 10.8% vs T, [50nM, 24 h] 209.6 ± 16.5%; DHT [10nM, 24h] 224.6 ± 8.6%; Figure 1E). In addition, *AR* overexpression increased lipid metabolism gene expression compared with cells transfected with vector alone (*FASN*: ctrl, 13.9 ± 2.0 vs *AR*, 66.8 ± 6.2; *ACC1*: ctrl, 1.0 ± 0.3 vs *AR*, 3.5 ± 0.3; *ACC2*: ctrl, 0.5 ± 0.1 vs *AR*, 1.0 ± 0.1; *CPT1*: ctrl, 1.8 ± 0.3 vs *AR*, 4.3 ± 0.2; *P* < .05).

Studies were also performed in primary cultures of human hepatocytes from both male and female donors. Both T and DHT were without effect in cultures from male patients; however, in samples from female donors, T increased lipogenesis (139.6 ± 17.6% [T, 5nM, 24 h] vs 100% [control]; *P* < .05). Interestingly, DHT decreased lipogenesis in hepatocytes from female donors only (Figure 1F).

### Regulation of lipid flux in human hepatocytes by glucocorticoids and insulin

**Figure 2.**
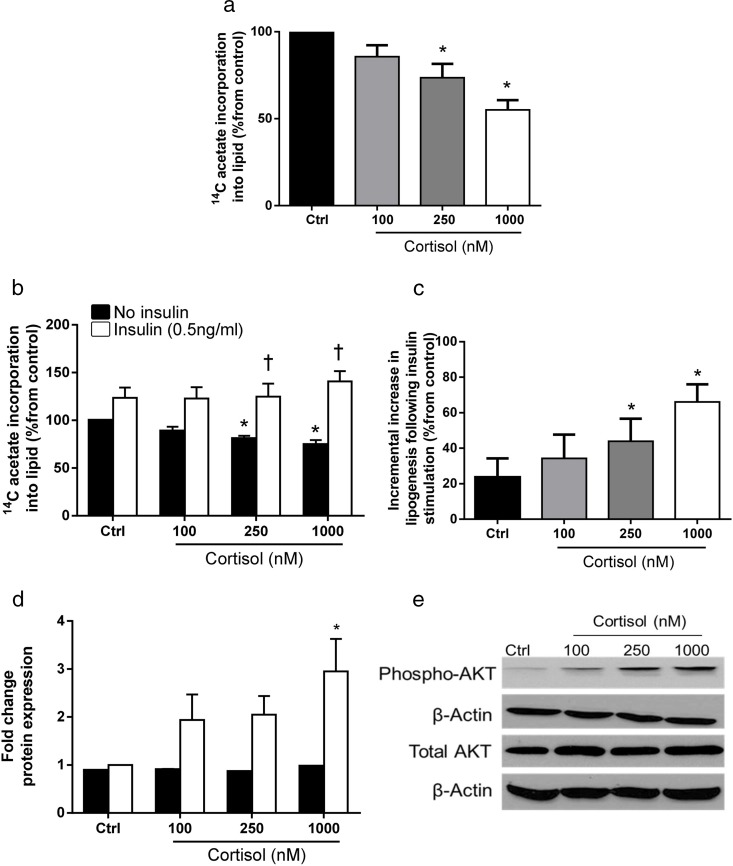
A, Cortisol decreases 1-[^14^C]-acetate incorporation into lipid in primary human hepatocytes from male donors. B, Whereas cortisol (black bars) decreases lipogenesis under hyperinsulinemic conditions (white bars), cortisol increases acetate incorporation into lipid. C, Cortisol treatment increases the ability of insulin to stimulate lipogenesis in a dose-dependent manner. D, Pretreatment of primary human hepatocytes from male donors with cortisol increases insulin-stimulated phosphorylation of AKT at residue ser473. The formal quantification of Western blot densitometry relative to β-actin (n = 4 experiments) is presented (total AKT [black bars] and pAKT ser473 [white bars]). E, Representative Western blot. Data presented are mean ± SE; *, *P* < .05.

*GC* receptor, *IRS1/2*, insulin receptor, and *AKT1/2* GC receptor, IRS1/2, insulin receptor, and AKT1/2 were all expressed in primary cultures of human hepatocytes from male donors. Incubation with cortisol alone or in combination with insulin did not alter gene expression levels (Supplemental Table 3). Cortisol decreased 1-[^14^C]-acetate incorporation into lipid in a dose-dependent manner (Figure 2A). Insulin increased lipogenesis in primary cultures of human hepatocytes (123.6 ± 10.7% [insulin, 5nM, 24 h] vs 100% [control]; *P* < .05) (Figure 2B). Interestingly, coincubation with increasing doses of cortisol increased the ability of insulin to simulate lipogenesis, suggesting that insulin and glucocorticoids may work synergistically to promote lipid storage in human hepatocytes (43.9 ± 12.7% [250 nM]; 66.13 ± 9.8% [1000 nM] vs control, (23.61 ± 10.7%); *P* < .05) (Figure 2C).

Cortisol treatment did not alter total PKB/akt levels. However, insulin-stimulated phosphorylation of PKB/akt at serine 473 increased following cortisol pretreatment in a dose-dependant manner (1.23-fold [100nM]; 1.68-fold [250nM]; 2.44-fold [1000nM] vs control, n = 4; *P* < .05) (Figure 2, D and E).

Cortisol treatment did not alter rates of β-oxidation of free fatty acid uptake in C3A cells (data not shown).

### *SRD5A2* regulates lipogenesis in human hepatocytes

The effects of T were similar to that of DHT upon lipogenesis in our hepatocyte models and we therefore postulated that *SRD5A2* may have a more important role to regulate GC exposure in this context.

**Figure 3.**
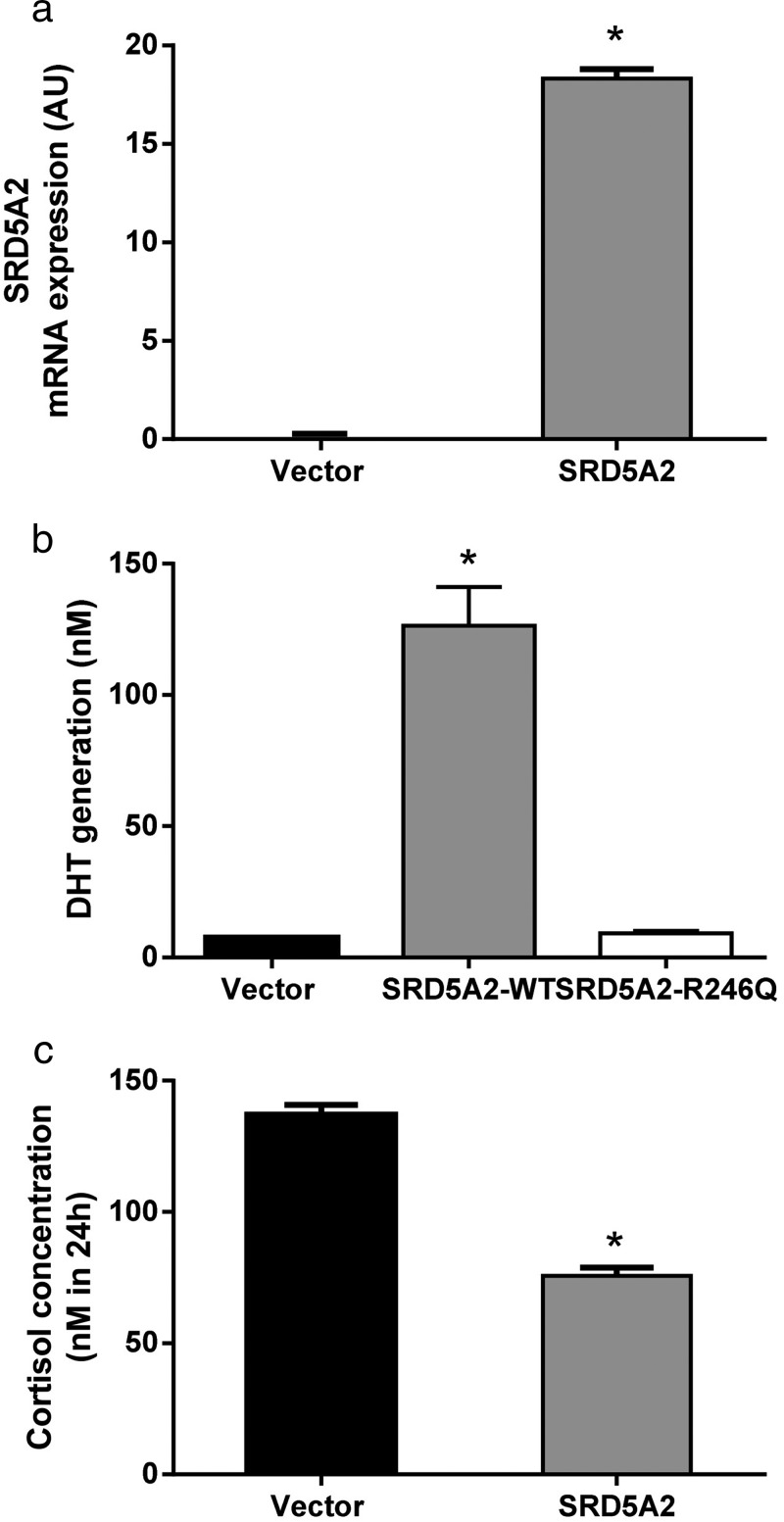
Increased *SRD5A2* expression following transfection into C3A cells. A, Transfection of wild-type *SRD5A2* (in contrast with the mutant R246Q construct) is associated with increased DHT generation from T (B) as well as clearance of cortisol (C). Data presented are mean ± SE of n = 3 experiments performed in triplicate; *, *P* < .05.

*SRD5A2* overexpression was confirmed using real-time PCR (Figure 3A). Functional activity was assessed through increased DHT generation following incubation with T (Figure 3B) and clearance of cortisol (Figure 3C) as measured by LC/MSMS and GC/MS, respectively. The mutant *SRD5A2* construct, R246Q, was without activity. Conversion of T to DHT was similar to that observed in the vector only transfection control (Figure 3B).

**Figure 4.**
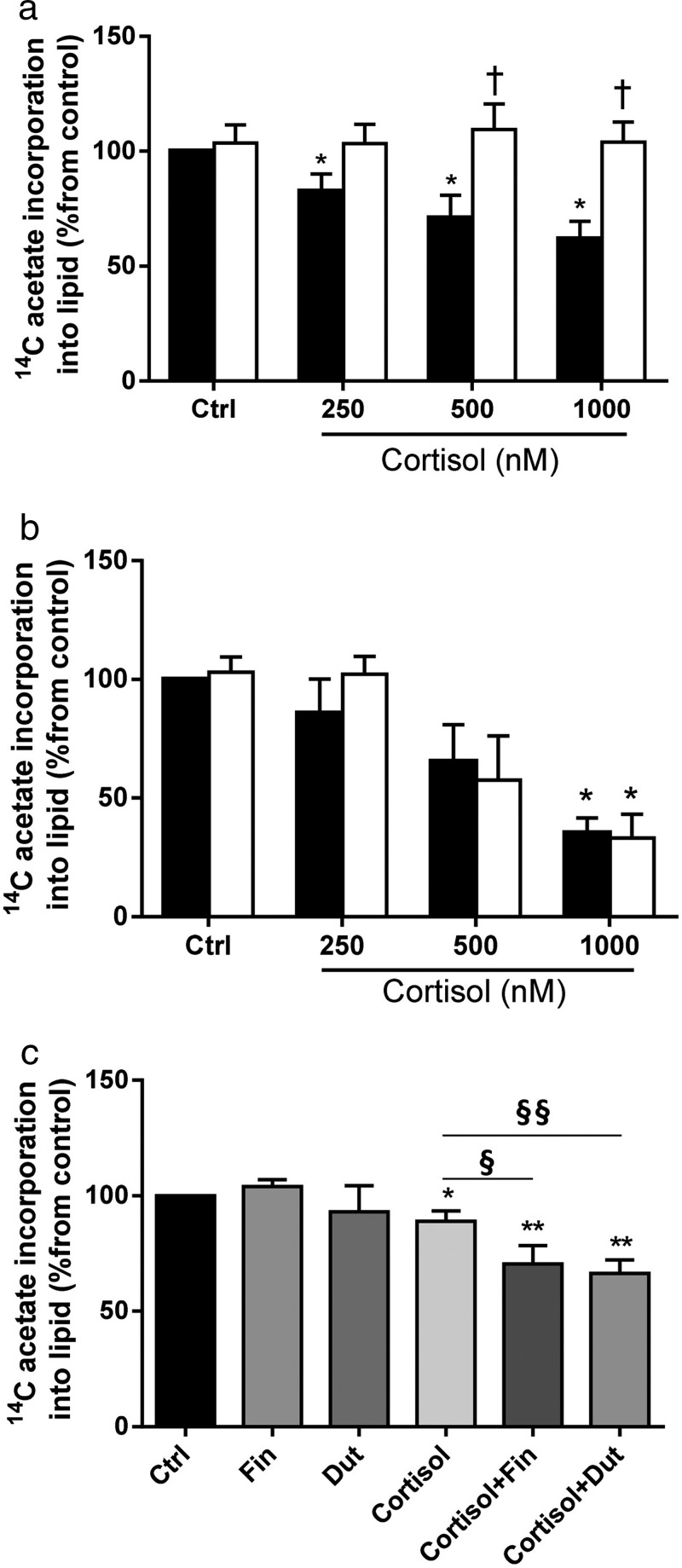
In C3A cells, the decrease in lipogenesis associated with increasing doses of cortisol (black bars; vector-only transfection) was abolished in cells transfected with wild-type *SRD5A2* (white bars) (A). Transfection of mutant R246Q *SRD5A2* was without effect (vector only, black bars; R246Q, white bars) (B). In primary cultures of human hepatocytes, pharmacological inhibition of *SRD5A2* with either the selective (finasteride) or nonselective inhibitor (dutasteride), augments the action of cortisol to suppress lipogenesis. Data presented are mean ± SE of n = 3–5 experiments performed in triplicate; *, *P* < .05; **, *P* < .*01* vs control; and §, *P* < .05; §§, *P* < .01 vs cortisol).

As observed previously, cortisol decreased lipogenesis in a dose-dependent manner in C3A cells transfected with vector construct alone and in the absence of cortisol, *SRD5A2* overexpression had no effect. However, in the presence of cortisol, *SRD5A2* restored lipogenesis to levels observed in untreated controls (eg, 61.9 ± 7.6%[cortisol] vs 103.8 ± 8.8% [*SRD5A2* + cortisol]; *P* < .05; control = 100%) (Figure 4A). Complementary experiments using the R246Q *SRD5A2* construct that is devoid of functional activity did not alter cortisol-mediated suppression of DNL (Figure 4B). To further endorse these findings, experiments were undertaken using pharmacological inhibitors of 5α-reductase isoforms in primary cultures of human hepatocytes. Consistent with our transfection studies, both finasteride (selective *SRD5A2* inhibitor) and dutasteride (nonselective *SRD5A1* and *2 SRD5A2* inhibitor) augmented the action of cortisol to suppress DNL (eg, 88.3 ± 5.3 vs 76.9 ± 5.2%, cortisol vs cortisol + finasteride; *P* = .05) (Figure 4C).

## Discussion

In this study, we have shown that androgens are able to increase lipid accumulation within human hepatocytes. Cross-sectional clinical studies have shown that low T concentrations are associated with increased hepatic steatosis in men (8, 9) and are consistent with findings in rodent models, suggesting that DHT treatment can decrease hepatic lipid accumulation (16). In contrast, women with polycystic ovary syndrome, a condition characterized by androgen excess as well as insulin resistance, are at an increased risk of developing NAFLD although the precise contribution of each of these processes (insulin resistance and androgen excess) to the development of NAFLD remains unclear (17, 18).

Although in vitro cell models are not able to replicate all the processes that contribute to the development of NAFLD in vivo, in C3A cells T and DHT increased lipid accumulation, but interestingly, in primary cultures, we observed sexually dimorphic effects. In cells derived from male donors, androgen treatment failed to have a significant effect upon lipogenesis; however, in female samples, 5nM T increased DNL. It is interesting to note that DHT did not alter lipogenesis in hepatocytes from male donors, but decreased lipogenesis in female hepatocytes. The mechanisms underpinning this observation and the physiological relevance (the concentrations of DHT used far exceed those seen in the female circulation) are not clear. The discrepancy between the effects of androgens on C3A cells and in primary cultures may reflect origin of C3A cells from human hepatoma [and the well described effect of androgens upon their pathogenesis (19)] and serves to emphasize the important of endorsing in vitro observations in additional models including human primary cultures.

Enhancing androgen action through androgen receptor overexpression increased DNL providing further evidence as to the potent ability of this pathway to regulate lipid accumulation. Interestingly, we observed no additional effects of providing additional *AR* ligand, perhaps suggesting maximal stimulation with receptor overexpression alone. Furthermore, *AR* overexpression alone in the absence of ligand was able to increase lipogenesis. Although it is possible that this may reflect existing intracellular androgen availability, ligand-independent activation of the *AR* remains plausible. This has been identified as a potential mechanism that might be crucial in regulating cell growth in the context of malignancy (20) notably in prostate cancer (21), although the precise mechanisms that underpin ligand-independent *AR* activation remain unclear. Importantly, not all actions of androgens upon the liver may be mediated by classical *AR* signaling. AR-independent regulation of lipogenesis in the liver has been observed in testicular feminized mice that lack a functional androgen receptor, with a reduction in lipogenesis following T treatment (22).

The effect of GCs to regulate carbohydrate metabolism in particular gluconeogenesis in the fasting state is well described. However, their effect on lipid metabolism remains relatively poorly understood in human models. We have previously shown in adipose and skeletal muscle that GCs decrease lipogenesis in the absence of insulin consistent with their role to mobilize fuel in the fasting state (23, 24). In the fed state, however, GCs and insulin act synergistically to drive lipid accumulation. Studies performed in rodent hepatocytes have demonstrated this relationship (25) and we have now shown this in primary cultures of human hepatocytes. In our in vitro model, the ability of GCs to enhance the ability of insulin to drive lipogenesis was associated with increased activation of the insulin-signaling cascade as demonstrated by increased phosphorylation of PKB/akt, similar to our published observations in human adipose tissue (26, 27). Although augmentation of insulin action by GCs has been observed in rodent hepatocytes (25), these in vitro data may not be reflective of more complex in vivo physiology. Clinical studies that have administered GCs have shown evidence of increased hepatic insulin resistance in most cases (28).

There is now an emerging role for the 5α-reductase isoforms in the regulation of metabolic phenotype. The ability of T to regulate lipid metabolism does not seem to be dependent upon the presence of *SRD5A2*. The C3A cell line does not express *SRD5A2*, and yet both T and DHT were able to stimulate lipogenesis to a similar extent. Our experiments therefore focused on the role of *SRD5A2* to regulate the effects of GCs upon liver metabolism. Although genetic ablation of *SRD5A1* in rodent models increases lipid accumulation and fibrosis, the precise mechanisms that underpin this are not clear (6, 7). In a recently published clinical study, nonselective 5α-reductase inhibition with dutasteride was associated with peripheral insulin resistance and it has been suggested that this may reflect a specific role for *SRD5A1* in skeletal muscle (29). The precise effect upon liver fat accumulation could not be determined as pre and postintervention assessment of hepatic lipid content was not performed.

The role of *SRD5A2* in clearing cortisol is well established through the examination of urinary steroid metabolite profiles in patients with proven *SRD5A2* mutations (5). Detailed metabolic studies in patients with mutations *SRD5A2* have not been performed. Increasing 5α-reductase activity is associated with an adverse metabolic phenotype (12, 30). This may reflect a compensatory mechanism to clear active GCs, in particular from the liver, in an attempt to protect it from lipid accumulation. With more severe disease activity decreases and this may increase GC exposure and may serve and a local anti-inflammatory measure to try to limit the progression of nonalcoholic steatohepatitis, fibrosis, and scarring (31).

In conclusion, we have demonstrated the potent actions of androgens and GCs to regulate lipid metabolism in human hepatocytes in vitro and shown that prereceptor regulation through that expression and activity of *SRD5A2* is able to modify their action. This has important implications not only in terms of predisposing individuals to the development of hepatic steatosis, but also to the large numbers of patients prescribed 5α-reductase inhibitors. Although the role of these compounds in the treatment of prostate-related disease is established, the long-term metabolic consequences of these medications have not been assessed.
